# Inhibition of gamma-secretase in Notch1 signaling pathway as a novel treatment for ovarian cancer

**DOI:** 10.18632/oncotarget.14152

**Published:** 2016-12-24

**Authors:** Zhaoyi Feng, Wandong Xu, Chenguang Zhang, Mengran Liu, Hongwu Wen

**Affiliations:** ^1^ Department of Obstetrics and Gynecology, Peking University First Hospital, Beijing 100034, China; ^2^ Center for Cancer Immunology and Cutaneous Biology Research Center, Massachusetts General Hospital, Harvard Medical School, Charlestown, MA 02129, USA; ^3^ Department of Medical Genetics, Capital Medical University, Beijing 100069, China

**Keywords:** epithelial ovarian carcinoma, Notch, Jagged1, NICD, γ-secretase

## Abstract

Epithelial ovarian cancer (EOC) is the leading cause of death for gynecological cancer. Most patients are not diagnosed until the cancer is at an advanced stage with poor prognosis. Notch1 signaling pathway plays an oncogenic role in EOC. There have been few studies on enzymatic activity of γ-secretase and the mechanism of how γ-secretase inhibitor works on cancer cell. Here, we show that Jagged1 and NICD were highly expressed in ovarian carcinoma. The expressions of Notch1, Jagged1 and NICD in Notch1 pathway did not correlate with outcome in ovarian cancer. The enzymatic activity of γ-secretase in ovarian cancer cell lines SKOV3, CAOV3 and ES2 is significantly higher than in normal ovarian epithelial cell line T29. DAPT (a γ-secretase inhibitor) reduced the enzymatic activity of γ-secretase, inhibited the proliferation, and increased the apoptosis in ovarian cancer cell lines. Hence, γ-secretase inhibitor may become a highly promising novel therapeutic strategy against ovarian cancer in the field of precision medicine.

## INTRODUCTION

Ovarian cancer is the most deadly gynaecological cancer, with approximately 200,000 new cases diagnosed globally each year and more than 150,000 deaths due to the disease annually [1]. Epithelial ovarian cancer (EOC) accounts for 90 % of all ovarian cancers and typically presents in post-menopausal women [2]. The pathologic type of EOC includes clear cell carcinoma, mucinous carcinoma, endometrioid carcinoma, low-grade serous ovarian carcinoma and high-grade serous carcinoma [3]. Most patients are not diagnosed until the cancer is at an advanced stage. Standard treatment for EOC involves cytoreductive surgery followed by platinum-based chemotherapy. Nevertheless, recurrence is frequent (around 70%) and prognosis is globally poor [4].

Notch signaling pathway is an evolutionarily conserved signaling pathway, which is composed of receptors, ligands and intracellular domain. In canonical Notch signaling, a Notch transmembrane receptor interacts extracellularly with a canonical Notch transmembrane ligand on a contacting cell, initiating proteolytic cleavage of the receptor by γ-secretase and the subsequent release of the intracellular domain (ICD) of the receptor. Notch intracellular domain (NICD) then translocates to the nucleus and plays its biological functions. Notch pathway plays essential roles in regulating cell differentiation, cell-cell communication, organ development and so on [5].

Notch1 and NICD were frequently expressed in ovarian cancer cell lines and specimens, concluding that Notch1 plays a role in ovarian cancer proliferation [6]. High expression of Notch1 and Jagged1 in breast cancer is linked to poor survival rates, and Jagged 1 is highly expressed in metastatic prostate cancer as compared to localized or benign prostatic tissue [7–9]. Nevertheless, the mechanism of how Notch receptor, ligand and NICD function in carcinogenesis of ovarian cancer is still unclear.

Gamma-secretase inhibitors have been developed to block Notch signaling and have entered clinical trials. These compounds inhibit γ-secretases that cleave Notch and additional proteins, inhibit the proteasome and can elicit endoplasmic reticulum stress [10–14]. Siemers ER et al first reported the effects of a γ-secretase inhibitor in a randomized study of patients with Alzheimer disease [15]. A multinational phase III clinical trial of LY450139 (a γ-secretase inhibitor) is currently under development as a disease-modifying therapy for Alzheimer disease [16]. Gamma-secretase inhibitor is also considered as a promising medicine for cancer therapy [17]. N-[N-(3,5-Difluorophenacetyl)-L-alanyl]-S-phenylglycine t-butyl ester (DAPT) is a γ-secretase inhibitor and commonly used to block Notch signaling [14]. Nethertheless, the enzymatic activity of γ-secretase in Notch1 signaling pathway and the mechanism of how γ-secretase inhibitor works on cancer cell are still unknown.

Here, we studied the expression of Notch1, Jagged1 and NICD in epithelial ovarian carcinoma tissues, analyzed the clinical significance and explored the potential anti-tumour effect of γ-secretase in epithelial ovarian carcinoma cell lines.

## RESULTS

### Jagged1 and NICD are highly expressed in ovarian carcinoma

**Figure 1:**
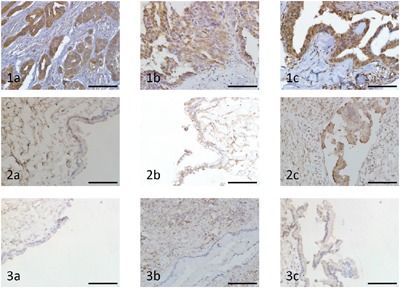
Immunohistochemical analysis of Notch1, Jagged1 and NICD expression in ovarian carcinoma and benign ovarian tumour (original magnification ×400, scale bar shows 50 μm) **1a.** High expression of Notch1 in ovarian cancer tissue. **1b.** High expression of Jagged1 in ovarian cancer tissue. **1c.** High expression of NICD in ovarian cancer tissue. **2a.** Low expression of Notch1 in benign ovarian tumour tissue. **2b.** Low expression of Jagged1 in benign ovarian tumour tissue. **2c.** Low expression of NICD in benign ovarian tumour tissue. **3a.** Negative expression of Notch1 in benign ovarian tumour tissue. **3b.** Negative expression of Jagged1 in benign ovarian tumour tissue. **3c.** Negative expression of NICD in benign ovarian tumour tissue.

We investigated the Notch1, Jagged1 and NICD immunohistochemical expression in all the 43 human ovarian cancer tissue specimens and 11 benign ovarian tumour tissue specimens. The percentage of expression of Notch1, Jagged1 and NICD in ovarian cancer specimens was 100% (43/43), 97.7% (42/43) and 100% (43/43) respectively, while the expression of Notch1, Jagged1 and NICD in benign ovarian tumour was detected in 10 (90.9%) specimens, 6 (54.5%) specimens and 1 (9.1%) specimen, respectively. There was not any significant different in the immunohistochemical scoring index (ICS) of Notch1 between ovarian cancer and benign ovarian tumour. Strikingly, the ICSs of Jagged1 and NICD in ovarian cancer were higher than in benign ovarian tumour (P < 0.01) (Table 1). Notch1 immunohistochemical expression was mostly confined to the cell membrane. Jagged1 immunohistochemical expression was mostly confined to the cell membrane and cytoplasm. NICD immunohistochemical expression was mostly confined to cytoplasm and nucleus (Figure 1).

**Table 1:** ICSs of Notch1, Jagged1 and NICD between ovarian cancer and benign ovarian tumour

Types	Number	Notch1	Jagged1	NICD
Ovarian cancer	43	6.7±2.2	5.3±2.4	5.3±2.3
Benign ovarian tumour	11	5.4±2.7	1.6±1.4	3.1±1.7
*P*		0.153^a^	<0.01^a^	<0.01^a^

a Mann–Whitney test.

We next analyzed the relationship between the expression of Notch1 pathway and clinicopathologic factors of ovarian cancer. The results presented that the expressions of Notch1, Jagged1 and NICD in ovarian cancers were not correlated with age, family history, ascites, serum CA125, size of tumour, FIGO stage, differentiation and pathology (P > 0.05) (Table 2).

**Table 2:** Relationship between the expression of Notch1, Jagged1 and NICD and clinicopathologic factors of ovarian cancer

	Number	Notch1 positive expression		*P*	Jagged1 positive expression		*P*	NICD positive expression		*P*
		Number	Percentage (%)		Number	Percentage (%)		Number	Percentage (%)	
Age				0.688			0.565			0.775^a^
<55	30	30	100		30	100		30	100	
≥55	13	13	13/13^c^		12	12/13^c^		13	13/13^c^	
Malignant cancer family history^a^				0.414			0.392			0.869^a^
Positive	32	32	100		31	97		32	100	
Negative	11	11	11/11^c^		11	11/11^c^		11	11/11^c^	
Ascites (ml)				0.306			0.968			0.660^a^
<1 000	28	28	100		27	96		28	100	
≥1 000	15	15	15/15^c^		15	15/15^c^		15	15/15^c^	
Serum CA_125_ (kU/L)				0.520			0.050			0.524^a^
<1 000	32	32	100		32	100		32	100	
≥1 000	11	11	11/11^c^		10	10/11^c^		11	11/11^c^	
Size of tumour (cm)				0.731			0.553			0.608^a^
<10	23	23	100		23	100		23	100	
≥10	20	20	100		19	95		20	100	
FIGO stage				0.095			0.861			0.772^b^
I	7	7	7/7^c^		6	6/7^c^		7	7/7^c^	
II	4	4	4/4^c^		4	4/4^c^		4	4/4^c^	
III	28	28	100		28	100		28	100	
IV	4	4	4/4^c^		4	4/4^c^		4	4/4^c^	
Histology				0.812			0.734			0.556^b^
Poorly differentiated	10	10	10/10^c^		10	10/10^c^		10	10/10^c^	
Moderately differentiated	25	25	100		25	100		25	100	
Highly differentiated	8	8	8/8^c^		7	7/8^c^		8	8/8^c^	
Pathology subtypes				0.757			0.205			0.186^b^
Serous carcinoma	31	31	100		30	97		31	100	
Mucinous carcinoma	4	4	4/4^c^		4	4/4^c^		4	4/4^c^	
Endometrioid carcinoma	5	5	5/5^c^		5	5/5^c^		5	5/5^c^	
Clear cell carcinoma	3	3	3/3^c^		3	3/3^c^		3	3/3^c^	

^a^ Mann–Whitney test.

^b^ Kruskal–Wallis test.

^c^ Number less than 20, not shown as percentage.

### Expressions of Notch1, Jagged1 and NICD do not correlate with outcome in ovarian cancer

We tested for the relationship between the expression of Notch1, Jagged1 and NICD and patient survival. Table 3 summarizes the association of Notch1, Jagged1 and NICD expression data with the patient's hazard ratio of survival. Patients with tumors expressing a high level of Notch1, Jagged1 and NICD were not more likely to die than patients with tumors expressing a moderate and low level of Notch1, Jagged1 and NICD (hazard ratio was 0.935, 1.236, and 1.104 respectively).

**Table 3:** Survival analysis of Notch1^Hi^/Notch1^Mo+Lo^, Jagged1^Hi^/Jagged1^Mo+L^^o^ and NICD^Hi^/NICD^Mo+L^^o^

Expression	n	Hazard ratio (95% CI)	*P*^a^
Notch1	43	0.935 (0.438-1.999)	0.863
Jagged1	42	1.236 (0.497-3.074)	0.656
NICD	43	1.104 (0.419-2.910)	0.842

95% CI 95% confidence interval, *lo* low, *mo* moderate, *hi* high.

^a^ Cox proportional hazard model.

**Figure 2:**
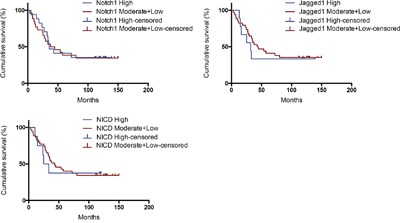
Kaplan–Meier analysis of patients with high vs. moderate+low expression of Notch1, Jagged1 and NICD

We next compared overall survival in patients whose tumors expressed high levels of Notch1, Jagged1 and NICD (No^Hi^, Ja^Hi^ and NI^Hi^) to patients with tumors expressing moderate and low levels of Notch1, Jagged1 and NICD (No^Mo+Lo^, Ja^Mo+Lo^ and NI^Mo+Lo^). Patients harboring tumors with No^Hi^, Ja^Hi^ and NI^Hi^ had similar 5-year survival rates compared with tumors with No^Mo+Lo^, Ja^Mo+Lo^ and NI^Mo+Lo^ (P > 0.05). (Table 4 and Figure 2).

**Table 4:** Comparison of tumors expressing high and moderate+low levels of Notch1, Jagged1 and NICD

Expression	n	5-year survival (%)	Mean survival time (mo) (95%CI)	*P*^a^
Notch1				
No^Mo+Lo^	26	46.15	40.5(24.3-.)	0.863
No^Hi^	17	41.18	34.9(23.1-.)	
Jagged1				
Ja^Mo+Lo^	33	45.45	42.1(27.1-.)	0.649
Ja^Hi^	9	33.33	31.5(12.7-.)	
NICD				
NI^Mo+Lo^	35	45.71	42.1(28.8-78.7)	0.842
NI^Hi^	8	37.50	28.6(10.2-.)	

95% CI 95% confidence interval, *lo* low, *mo* moderate, *hi* high.

^a^ Log rank test.

### The enzymatic activity of γ-secretase in ovarian cancer cell lines is significantly higher than in normal ovarian epithelial cell line

**Figure 3:**
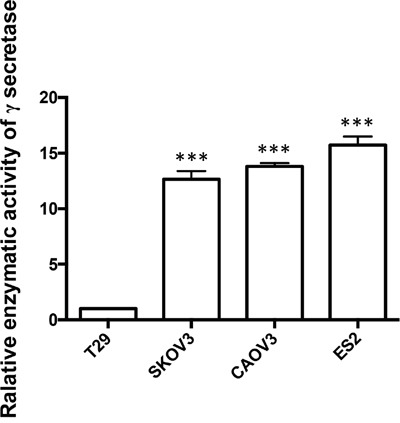
The relative enzymatic activity of γ-secretase in SKOV3, CAOV3, ES2 and T29 cell lines The relative enzymatic activity of γ-secretase of T29 was set as 1. *** p < 0.001.

In order to confirm if γ-secretase in Notch1 pathway plays a role in the carcinogenesis of ovarian cancer, we detected the relative enzymatic activity of γ-secretase by dual-luciferase reporter assay system. The activity of γ-secretase in T29 was supposed as 1.00. The relative activity of γ-secretase in SKOV3, CAOV3 and ES2 was 12.60, 13.80 and 15.73, respectively, which was significantly higher than the activity of γ s-secretase in T29. (Figure 3)

### DAPT reduces the enzymatic activity of γ-secretase in ovarian cancer cell lines

**Figure 4:**
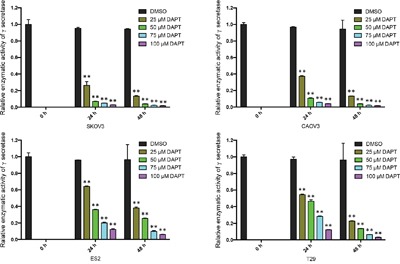
The relative enzymatic activity of γ-secretase in ovarian cancer cell lines and normal ovarian epithelial cell line after treatment of DAPT The relative enzymatic activity of γ-secretase at 0 h was set as 1 in each group. ** p < 0.01.

In order to determine if DAPT can reduce the enzymatic activity of γ-secretase in ovarian cancer cells and benign ovarian epithelial cell, we tested the enzymatic activity of γ-secretase in SKOV3, CAOV3, ES2 and T29 after DAPT treatment with different concentrations and different time. Interestingly, after 25–100 μM of DAPT was added for 24 h or 48 h, the enzymatic activity of γ-secretase in SKOV3, CAOV3 and ES2 cell lines declined significantly in a dose- and time-dependent manner (P < 0.01). Also, the enzymatic activity of γ-secretase in T29 cell line declined after DAPT treatment. (Figure 4)

### DAPT inhibits the proliferation of ovarian cancer cell lines

**Figure 5:**
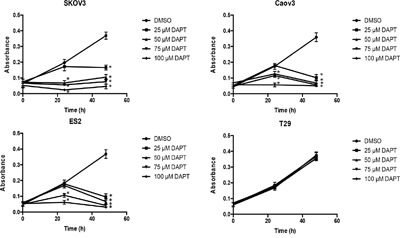
Effect of different concentrations of DAPT on the proliferation of ovarian cancer cell lines and normal cell line * p < 0.05.

Next, in order to determine if DAPT exhibits inhibitory effect on ovarian cancer cell proliferation, we investigated the role of DAPT in proliferation of ovarian cancer cell lines. After SKOV3, CAOV3 and ES2 cells were treated with 25–100 μM DAPT for 24 h or 48 h, an MTT assay was performed to detect cell proliferation. MTT data showed that DAPT (50 μM, 75 μM and 100 μM) inhibited the proliferation of SKOV3 and CAOV3 cell lines 24 h and 48 h after DAPT treatment compared with control group (P < 0.05). DAPT (75 μM and 100 μM) 24 h and DAPT (25 μM, 50 μM, 75 μM and 100 μM) 48 h after treatment inhibited the proliferation of ES2 cell line compared with control group (P < 0.05). DAPT (25 μM) inhibited the proliferation of SKOV3, CAOV3 and ES2 cell lines only 48 h after DAPT treatment (P < 0.05). However, DAPT (25–100 μM) showed no inhibitory effect on the proliferation of T29 cell line at 24 h or 48 h after DAPT treatment (P > 0.05). (Figure 5)

### DAPT increases the apoptosis of ovarian cancer cell lines

**Figure 6:**
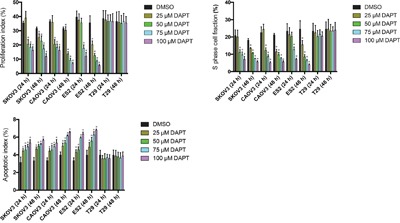
Effect of different concentrations of DAPT on the PI, SPF and AI of ovarian cancer cell lines and normal cell line * p < 0.05.

To test the effect of DAPT on ovarian cancer cell apoptosis, flow cytometry was performed. It is found that DAPT decreased the PI and SPF and increased the AI in SKOV3, CAOV and ES2 cell lines as concentration of DAPT increased and time after DAPT treatment prolonged. The initiation concentration of DAPT decreased the PI and SPF in SKOV3, CAOV3 and ES2 cell lines was 50 μM, 50 μM and 75 μM at 24 h after DAPT treatment (P < 0.05). The initiation concentration of DAPT increased the AI in SKOV3, CAOV3 and ES2 cell lines was all 25 μM at 24 h after DAPT treatment (P < 0.05). However, DAPT (25–100 μM) did not change the PI, SPF and AI in T29 cell line at 24 h or 48 h after DAPT treatment (P > 0.05). (Figure 6)

## DISCUSSION

In 1991, Ellen LW et al found that Notch gene was broken by chromosomal translocation in T lymphoblastic neoplasms, which opened a new era of Notch signaling and malignancies [18]. Rose SL et al found abundant NICD expression in ovarian cancer cell lines, as well as in 16 of 21 (76%) human ovarian cancer samples. The expression of NICD was greatly reduced in ovarian cancer cell lines after treatment with Notch1 siRNA. Furthermore, depletion of Notch1 led to growth inhibition of ovarian cancer cell lines, which provide evidence for target therapy of ovarian cancer by inhibiting Notch pathway [6]. However, Hopfer O et al concluded that Notch1 pathway was expressed in ovarian adenomas and cancers and the transcription factor hairy and enhancer of split 1 (HES1) was strongly expressed in ovarian cancers [19]. In this research, we found that Notch1 was widely expressed in both epithelial ovarian cancers and benign tumours, while the ICSs of Jagged1 and NICD in ovarian cancer were higher than in benign ovarian tumour. A possible reason is that Notch signaling pathway is an evolutionarily conserved signaling pathway. The combination of ligand and receptor activated Notch signaling and receptor was cleaved into NICD by γ-secreatase. NICD interacted with HES1 or other signaling such as Ras and PI3K/Akt and played an oncogenic role in ovarian cancer [20, 21]. Notch pathway cannot be triggered as carcinogenesis without the combination of ligand and Notch receptor.

Parr C et al found that Notch1 expression was low in grade 1 breast cancers and increased in poorly-differentiated breast cancers [22]. Wang M et al found that expression of Notch1 increased gradually with the poor differentiating of cancer tissues and the increasing of FIGO stage in ovarian cancer tissues [23]. In this research, we found the expressions of Notch1, Jagged1 and NICD in ovarian cancers were not correlated with clinicopathologic factors, which needs more specimens to be demonstrated.

Lin JT et al found that high-level coexpression of Notch1 and Jagged1 was associated with poor overall survival in patients with head and neck cancer [24]. Wu K et al found that high Jagged1 expression was statistically linked to reduced overall and disease-free survival in clear cell renal cell carcinoma patients, especially at the early stage [25]. Xu X et al concluded that the activation of Notch1 pathway might indicate a poor prognosis in acute myeloid leukemia. Especially, Notch1, Jagged1 and Delta1 expression might be relevant prognostic markers in intermediate risk acute myeloid leukemia [26]. In our research, we did not find a correlation between expressions of Notch1, Jagged1 and NICD and survival of ovarian cancer patients, although there seems to be a tendency that high expression of Jagged1 may have a poor survival (Figure 2). A possible reason is that the number of ovarian cancer patients included in this research is low (n = 43). In future study, in order to obtain more significant result, the number of cancer patients should be increased and the time of follow-up should be prolonged.

Gamma secretase is the key element in Notch pathway and the oncogenic role of Notch signaling relies on the cleavage of Notch receptor by γ-secretase. We haven't found previous report on detecting the enzymatic activity of γ-secretase in Notch pathway related ontogenesis. In this research, the enzymatic activity of γ-secretase was detected by Dual Luciferase Reporter Assay. Plasmid Notch1 ΔE-GVP expresses Notch1 plus transcription activator Gal4-VP16. Plasmid MH100 expresses luciferase and is used as a reporter. In the presence of γ-secretase, Notch1 ΔE-GVP plus Gal4-VP16 is cleaved into intracellular domain plus Gal4-VP16 and translocates to the nucleus and activates transcription from a UAS promoter element. Luciferase expressed by MH100 is activated by USA promoter element [27–29]. We found that the enzymatic activity of γ-secretase in ovarian cancer cell was significantly higher than in normal ovarian epithelial cell, which demonstrates γ-secretase gets involved in the carcinogenesis of ovarian cancer. We firstly used Dual Luciferase Reporter Assay to detect enzymatic activity of γ-secretase, which could be widely used in cancer research. The therapeutic effect of γ-secretase inhibitor was also demonstrated on DAPT treated ovarian cancer cells. Chen X et al reported sequential combination therapy of ovarian cancer with cisplatin and γ-secretase inhibitor MK-0752 in cell and mice models [30]. In this research, we found that DAPT had therapeutic effect by inhibiting proliferation and increasing apoptosis of ovarian cancer cells. Interestingly, DAPT showed no effect on proliferation and apoptosis of the normal ovarian surface epithelial cell line T29 although DAPT reduced its enzymatic activity of γ-secretase, possibly because Notch pathway may not be essential for normal ovarian epithelial cells. Presumably, DAPT may minimize the side effect on normal ovarian tissue. Therefore, γ-secretase inhibitor may become a highly promising novel experimental therapeutic strategy against ovarian cancer in the field of precision medicine.

## MATERIALS AND METHODS

### Patients and specimens

Forty-three human ovarian tissue specimens were obtained from patients who underwent surgical resection of ovarian from the Department of Obstetrics and Gynecology of Peking University First Hospital, Beijing, China, between March 2004 and July 2007. At the time of surgery, no patient had received chemotherapy or radiotherapy. Histomorphology of all specimens was confirmed by the Department of Pathology, Peking University First Hospital. Tumours were staged according to the Federation International of Gynecology and Obstetrics (FIGO) staging of ovarian cancer in 2014. Benign epithelia ovarian tumour tissues were obtained from patients undergoing surgery for benign ovarian cyst. A total of 54 specimens were analyzed, including 43 ovarian epithelial carcinoma tissues, and 11 benign ovarian epithelial tumour tissues. The ovarian cancer patients were followed up for a median 63.8 months (range 2.9–250.1 months) and ended at July 5 2016.

### Immunohistochemistry

Expression of Notch1, Jagged1 and Notch1 ICD in the pathologic sections was detected by an immunoperoxidase method. The paraffin-embedded ovarian tissue sections were deparaffinized with xylene. Peroxidase activity was quenched using 3.0% hydrogen peroxide. The antigen retrieval was by microwaving sections in 0.01 M sodium citrate, pH 6.0. Sections were incubated overnight with goat anti-human Notch1 (1:100; Santa Cruz), goat anti-human Jagged1 (1:100; Santa Cruz) and rabbit anti-human NICD (1:100; Millipore) at 4°C. The color development was using ABC kit.

ICS was then calculated by multiplying the intensity and percentage scores to determine the following results: 0, 1, 2, 3, 4, 6, and 9. In statistical analyses, ICS of 0 and 1 were defined as negative (-), ICS of 2 and 3 were defined as low expression (+), ICS of 4 and 6 were defined as mediate expression (++), and ICS of 9 was defined as high expression (+++) [31, 32].

### Cell culture

Human ovarian adenocarcinoma cell lines cell line SKOV3 and human clear ovarian cancer cell line ES2 were cultured in RPMI1640 medium with 10 % fetal bovine serum. Human ovarian adenocarcinoma cell line CAOV3 was cultured in DMEM with 10 % fetal bovine serum. Human immortalized ovarian surface epithelial cell line T29 was cultured in Medium 199 and MCDB 105 Medium. All media contained 1% penicillin and streptomycin. All cell lines were kindly provided by Center of Gynecologic Oncology, Peking University People's Hospital, Beijing, China.

### Dual luciferase reporter assay system

A Dual-Luciferase Reporter Assay (Promega) was used to detect the relative enzymatic activity of γ-secretase. SKOV3, CAOV, ES2 and T29 cells were seeded in 96-well plates at 10^5^ cells per well. Then the cells were transfected respectively with 200ng MH100, 100ng Notch1 ΔE-GVP and 2ng pRL-CMV using Lipofectamine 2000 (Invitrogen, Carlsbad, CA, USA) according to the manufacturer's protocol. The experiment was independently repeated at least three times. The transfected cells were collected in lysis buffer. Luciferase activity in triplicate samples for each condition was measured with a luminometer. Renilla luciferase activity was used to normalize the firefly luciferase activity.

### MTT assay

An MTT assay was performed to assess the effect of DAPT on cell proliferation. Briefly, the cells were added to 96-well plates and then treated with DAPT (25–100 μM, Sigma-Aldrich) and DMSO (Sigma-Aldrich) for 24–48 h. Next, the MTT reagent was introduced to each well, and the supernatants were removed 4 h later. A total of 150 μL DMSO was used to dissolve the resultant formazan crystals. The absorption was read at 490 nm using a spectrophotometer.

### Flow cytometry

Cells were added treated with DAPT (25–100 μM) and DMSO for 24–48 h respectively and analyzed by flow cytometry. S phase cell fraction (SPF) was calculated according to the equation: SPF=S/(S+G1+G2)×100%. Proliferation index (PI) was calculated according to the equation: PI=(S+G2)/ (S+G1+G2)×100%. Apoptosis was presented as apoptotic index (AI), which is expressed as the ratio of apoptotic cells to all the cells checked as a percentage.

### Statistical analysis

A comparison of protein expression was evaluated with Mann-Whitney test. A comparison of clinicopathologic characteristics was evaluated with the Mann-Whitney test and Kruskal-Wallis test. The survival rate was calculated by the Kaplan-Meier method. The Cox proportional hazard regression model was used to determine the joint effects of several variables on survival. The differences between the survival curves were tested by the log rank test. The activity of enzymatic activity of γ-secretase, cell proliferation and apoptosis were analyzed by *t* test. All statistical analyses were performed with SPSS for Windows version 22.0 (SPSS, Chicago, IL, USA).
